# Cholera outbreak in Homa Bay County, Kenya, 2015

**DOI:** 10.11604/pamj.supp.2017.27.1.12563

**Published:** 2017-05-28

**Authors:** Jane Njoki Githuku, Waqo Gufu Boru, Casey Daniel Hall, Zeinab Gura, Elvis Oyugi, Rogath Saika Kishimba, Innocent Semali, Ghada Nadim Farhat, Meeyoung Mattie Park

**Affiliations:** 1 Kenya Field Epidemiology and Laboratory Training Program, Ministry of Health, Kenya; 2 Rollins School of Public Health, Emory University, Atlanta, USA; 3 Tanzania Ministry of Health and Social Welfare, Tanzania; 4 Tanzania Field Epidemiology and Laboratory Training Program, Tanzania; 5 Muhimbili University of Health and Allied Sciences, Dar es Salaam, Tanzania

**Keywords:** Public Health, epidemiology, cholera, re-emerging disease, outbreak investigation

## Abstract

Cholera is among the re-emerging diseases in Kenya. Beginning in December 2014, a persistent outbreak occurred involving 29 out of the 47 countries. Homa Bay County in Western Kenya was among the first counties to report cholera cases from January to April 2015. This case study is based on an outbreak investigation conducted by FELTP residents in Homa Bay County in February 2015. It simulates an outbreak investigation including laboratory confirmation, active case finding, descriptive epidemiology and implementation of control measures. This case study is designed for the training of basic level field epidemiology trainees or any other health care workers working in public health-related fields. It can be administered in 2-3 hours. Used as adjunct training material, the case study provides the trainees with competencies in investigating an outbreak in preparation for the actual real-life experience of such outbreaks.

## How to use this case study

**General instructions:** this case study should be used as adjunct training material for novice epidemiology trainees to reinforce the concepts taught in prior lectures. The case study is ideally taught by a facilitator in groups of about 20 participants. Participants are to take turns reading the case study, usually a paragraph per student. The facilitator guides the discussion on possible responses to questions. The facilitator may make use of flip charts to illustrate certain points. Additional instructor’s notes for facilitation are coupled with each question in the instructor’s guide to aid facilitation.

**Audience:** this case study was developed for novice field epidemiology students. These participants are commonly health care workers working in the county departments of health whose background may be as medical doctors, nurses, environmental health officers or laboratory scientists who work in public health-related fields. Most have a health science or biology background.

**Prerequisites:** before using this case study, participants should have received lectures on disease surveillance and outbreak investigation.

**Materials needed:** flash drive, flip charts, markers, computers with MS Excel

**Level of training and associated public health activity:** Novice – Outbreak investigation

**Time required:** 2-3 hours

**Language:** English

## Case study material

- Download the case study student guide (PDF - 1.69 MB)
- Request the case study facilitator guide.

## References

[1] County Government of Homa bay (2013). County Government of Homa bay “A County of Choice” First County Integrated Development Plan.

[2] CDC (2011). Differential diagnosis: outbreaks of acute watery diarrhea. Global Disease Detection (GDD) Manual “Rapid Diagnostic Tests for Epidemic Diseases” 2011 (draft).

[3] Dawson AL, Ailes EC, Gilboa SM, Simeone RM, Lind JN, Farr SL (2016). Antidepressant Prescription Claims Among Reproductive-Aged Women With Private Employer-Sponsored Insurance - United States 2008-201 MMWR.

[4] World Health Organization (2012). Centers for Disease Control and Prevention and Ministry of Health. Technical Guidelines for Integrated Disease Surveillance and Response in Kenya.

